# Characterization and efficacy against carbapenem-resistant *Acinetobacter baumannii* of a novel *Friunavirus* phage from sewage

**DOI:** 10.3389/fcimb.2024.1382145

**Published:** 2024-04-22

**Authors:** Zhitao Wang, Xue Yang, Hui Wang, Shuxian Wang, Ren Fang, Xiaotian Li, Jiayin Xing, Qianqian Wu, Zhaoli Li, Ningning Song

**Affiliations:** ^1^Weifang Key Laboratory of Respiratory Tract Pathogens and Drug Therapy, School of Life Science and Technology, Shandong Second Medical University, Weifang, China; ^2^School of Pharmacy, Shandong Second Medical University, Weifang, China; ^3^Department of Clinical Laboratory, Affiliated Hospital of Shandong Second Medical University, Weifang, China; ^4^SAFE Pharmaceutical Technology Co. Ltd., Beijing, China

**Keywords:** *Acinetobacter baumannii*, phage Ab_WF01, crab, phage therapy, *Galleria mellonella*, mice

## Abstract

Carbapenem-resistant *Acinetobacter baumannii* (CRAB) has become a new threat in recent years, owing to its rapidly increasing resistance to antibiotics and new effective therapies are needed to combat this pathogen. Phage therapy is considered to be the most promising alternative for treating CRAB infections. In this study, a novel phage, Ab_WF01, which can lyse clinical CRAB, was isolated and characterized from hospital sewage. The multiplicity of infection, morphology, one-step growth curve, stability, sensitivity, and lytic activity of the phage were also investigated. The genome of phage Ab_WF01 was 41, 317 bp in size with a GC content of 39.12% and encoded 51 open reading frames (ORFs). tRNA, virulence, and antibiotic resistance genes were not detected in the phage genome. Comparative genomic and phylogenetic analyses suggest that phage Ab_WF01 is a novel species of the genus *Friunavirus*, subfamily *Beijerinckvirinae*, and family *Autographiviridae*. The *in vivo* results showed that phage Ab_WF01 significantly increased the survival rate of CRAB-infected *Galleria mellonella* (from 0% to 70% at 48 h) and mice (from 0% to 60% for 7 days). Moreover, after day 3 post-infection, phage Ab_WF01 reduced inflammatory response, with strongly ameliorated histological damage and bacterial clearance in infected tissue organs (lungs, liver, and spleen) in mouse CRAB infection model. Taken together, these results show that phage Ab_WF01 holds great promise as a potential alternative agent with excellent stability for against CRAB infections.

## Introduction

*Acinetobacter baumannii* is one of the most challenging hospital-acquired pathogens worldwide and causes a wide spectrum of infections, including bacteremia, pneumonia, skin and soft tissue infections, and meningitis (Ibrahim et al., 2021). As a member of the ESKAPE (*Enterococcus faecium*, *Staphylococcus aureus*, *Klebsiella pneumoniae*, *Acinetobacter baumannii*, *Pseudomonas aeruginosa*, and *Enterobacter* species) group of pathogens, *A. baumannii* is considered a worldwide threat because it tends to acquire multidrug resistance at previously unforeseen rates (Rai and Kumar, 2022). Over the past few decades, carbapenems have been recognized as the most effective antibiotics for combating infections caused by multidrug-resistant *A. baumannii*. To date, *A. baumannii* is resistant to most clinically common antibiotics, and polymyxin B is considered the last-resort antibiotic option for treating infections caused by carbapenem-resistant gram-negative bacteria (Nang et al., 2021). However, polymyxin B resistance genes have also been reported in *A. baumannii*, which results in infections caused by carbapenem-resistant *A. baumannii* (CRAB) that are difficult to treat due to the lack of drugs available in contemporary clinical practice (Martins-Sorenson et al., 2020). It is therefore imperative that new antibiotics and alternative treatment strategies be developed in order to combat the serious threat posed by CRAB infections (Vukotic et al., 2020).

Phages are natural bacterial killers that are abundant in the biosphere and infect specific bacteria. Since their discovery, phages have been used in practical applications, but the rise of antibiotics has led to the decline of phage therapy (Taati Moghadam et al., 2020). Due to emerging and increasing bacterial resistance to antibiotics worldwide, phages have attracted considerable attention as a promising alternative to antibiotics to combat antimicrobial pathogen challenges. In recent years, phages have shown great therapeutic potential for treating infections caused by *A. baumannii* (Zhang et al., 2022), such as *Acinetobacter* phage have been reported for the successful therapy of multidrug-resistant (MDR) *A. baumannii* infection in diabetic patients (Schooley et al., 2017). More and more *in vivo* study confirmed that phages have significant therapeutic effect against multidrug-resistant *A. baumannii* strain without deleterious side effects (Hua et al., 2018; Torabi et al., 2021; Sisakhtpour et al., 2022). Moreover, researchers have studied lytic phages of multidrug-resistant (MDR) *A. baumannii* and found that it is expected to become an alternative therapy in hospital clinical practice (Chu et al., 2022). However, the emergence of phage-resistant bacteria has also occurred in recent years, which is a major limitation in phage therapy (Oechslin, 2018). The genomic sequencing analysis of clinically isolated *A. baumannii* by Ambroa et al. suggested that the number of genes associated with phage resistance may be rising year by year (Ambroa et al., 2022). Therefore, it is important to isolate new phages and characterize individual phages from experiments *in vitro* and *in vivo* (Rai and Kumar, 2022).

In this study, we isolated and characterized a novel lytic phage infecting *A. baumannii*, Ab_WF01 from wastewater at the Affiliated Hospital of Shandong Second Medical University. The morphology, biological characterization, and complete genomic analysis of the phage were performed. Moreover, *in vivo* the efficacy of phage Ab_WF01 against CRAB infection both *Galleria mellonella* model and a mouse model were evaluated, in order to provide a theoretical foundation for the practical application of bacteriophages in the battle against CRAB infections.

## Materials and methods

### Animals

Six to eight-week-old C57BL/6j mice (random male females) were purchased from Ji’nan Pengyue Laboratory Animal Breeding Co., Ltd.

### Bacterial strains and growth conditions

Resistant clinical isolates of *A. baumannii*, *Escherichia coli* (*E. coli*), Enterococcus faecalis (*E. faecalis*), Methicillin-resistant *Staphylococcus aureus* (MRSA), and Pseudomonas aeruginosa (*P. aeruginosa*) strains were provided by the clinical laboratory at the Affiliated Hospital of Shandong Second Medical University, Shandong Province, China. Bacterial isolates were obtained from the blood of inpatients, and samples were then spread on Columbia Blood Agar Plate (CBAP; mlbio, Shanghai, China) and incubated overnight at 37°C. One colony on CBA was transferred to 5 mL of Lysogeny Broth (LB) and then incubated at 37°C with shaking at 220 rpm. The 16S rRNA gene was amplified by PCR using universal bacterial primers F27 and R1492 to identify the strain (Sangon Biotech Co., Ltd., Shanghai, China). Sequence identification was performed using the Basic Local Alignment Search Tool (BLAST) of the NCBI database. To maintain the bacteria for long-term storage, LB broth containing 20% glycerol was used and maintained at -80°C.

### Phage isolation and purification

Wastewater samples collected from sewers around the Affiliated Hospital of Shandong Second Medical University were centrifuged to remove debris at 5000 g for 10 min and filtered through a 0.22 μm microporous membrane (Biosharp Biotechnology, China) to remove bacterial cells. CRAB was cultured and adjusted to a final OD_600_ of 0.6 (1 x 10^8^ CFU/mL). Ten milliliters of sewage filtrate were supplemented with 10 mL of LB, and the mixture was incubated with 200 μL of CRAB. After overnight incubation at 37°C, the culture solution was centrifuged at 5000 g for 10 min and filtered using a 0.22 μm syringe sterile filter. Initial phage isolation was used to conduct spot tests using the CRAB strain, and the presence of plaques was then observed. Phages were purified at least three times using the double-layer agar method until a one-plaque morphology was observed.

### Host range of phage

The host range of the purified phage was determined by spot tests, as previously described (Kutter, 2009) on six other resistant clinical isolates, including multi-drug resistant (MDR) *Enterococcus faecium*, MRSA, *Klebsiella pneumoniae*, CRAB, Carbapenem-Resistant *Pseudomonas aeruginosa* (CRPA), and Carbapenem-Resistant *Escherichia coli* (CREC). Briefly, each exponential phase *A. baumannii* clinical isolate was mixed with melting 0.7% LB agar and then quickly poured onto an LB agar plate. After solidification, 10 μL of diluted phage solution (10^6^ PFU/mL) was plated onto each of the bacterial lawns, and a clear zone for bacterial lysis was observed after overnight incubation at 37°C. The experiment was repeated three times, and two parallel samples were collected each time.

### Transmission electron microscopy

The morphology of the concentrated phages (10^11^ PFU/mL) was examined by TEM. Phage Ab_WF01 was dropped attached to carbon-coated copper grids and negatively stained with 2% phosphotungstic acid for 5 minutes. Phage morphology was observed using a transmission electron microscope (Hitachi HT7700, Tokyo, Japan) at 80 kV.

### Optimal multiplicity of infection

To determine the phage optimal multiplicity of infection (MOI), a series of ten-fold dilutions of the phage were prepared in SM buffer. One hundred microliters of the phage were mixed with 100 μL of CRAB using a conventional double-layer agar method and incubated overnight at 37°C. The plaques were counted and calculated as plaque-forming units per milliliter (PFU/mL).

### One-step growth curve

For the one-step growth assay, host bacteria were infected with phages at an MOI of 0.01, and allowed to adsorb the phages for 15 min. The sample was centrifuged at 12,000 rpm for 10 min to remove the supernatant, and the precipitate was washed twice with LB. The pellets were suspended in 20 mL LB, followed by incubation at 37°C and 220 rpm. Samples were collected at 10 min intervals for up to 120 min using the double-layer method to determine the phage titer. Previously described methods were used to calculate the latency period and burst size (Kropinski, 2018).

### Thermal and pH stability assay

For thermal stability testing of phage Ab_WF01, 1 mL of phage was incubated in five different microtubes at 25°C, 37°C, 50°C, 60°C, 70°C, and 80°C for 1 h in a water bath. The phage samples were allowed to incubate for 1 h at various temperatures, and then they were chilled on ice to gradually cool down. The stability and vitality of the phage were determined by means of a double-layer agar assay.

To test the effects of pH on the stability of phage Ab_WF01, the pH of LB broth in different pH values was adjusted from 1 to 14 using 1 M sulfuric acid and 1 M sodium hydroxide. Five hundred microliters of phage were added to each tube and incubated at 37°C for 1 h. Phage samples were neutralized to pH 7 by adding sulfuric acid (1 M) and sodium hydroxide (1 M), and then all tubes were filtered using a 0.45 μm syringe filter to prevent any contamination before the double-layer agar assay.

### Chloroform sensitivity assay

Phage capsids generally contain lipids. To test the chloroform tolerance of the phage Ab_WF01, 1 mL of the phage suspension (10^9^ PFU/mL) was incubated with various volumes of chloroform (0%, 1%, 2%, or 5%, w/v) in tubes at 37°C for 30 min. The titers of the mixtures were determined using a double-layer agar assay.

### Host cell lytic activity assay

To investigate adsorption kinetics, the host bacterium (CRAB) was cultured in an LB medium and infected with phages at an MOI of 0.01. During shake culture at 37°C, 1 mL sample were taken at 1 h intervals for 12 h, and bacterial counts were calculated by a single plate-serial dilution method. The tests were repeated in triplicate.

### Complete genome sequencing and bioinformatics analysis

The genomic DNA of Ab_WF01 was extracted from the phage stock using a TIANamp Virus DNA/RNA Kit (Tiangen, Beijing, China), according to the manufacturer’s instructions. Whole genome sequencing was performed using the Illumina sequencing platform at Sangon Biotech Co., Ltd. (Shanghai, China). The SPAdes software was used for *de novo* genome assembly (Bankevich et al., 2012). The open reading frames (ORFs) were annotated by Rapid Annotation using Subsystem Technology v2.0 (RAST) (Aziz et al., 2008). The prediction of tRNAs in genomic sequences was predicted using tRNAscan-SE (Chan and Lowe, 2019). Virulence factors and antibiotic resistance genes of the phage genome were compared with those in the VFDB database (http://www.mgc.ac.cn/VFs/main.htm), and CARD database (https://card.mcmaster.ca/). A circular map of the phage genome was constructed using the CGview Server (Grant and Stothard, 2008). For whole-genome analysis, the genome sequence was aligned and compared with that of other phages using BLASTn, and the phage genomes of Ab_WF01 and vB_AbaP_46-62_Aci07 were compared using the Artemis Comparison Tool (Carver et al., 2005). The complete genome sequence of phage Ab_WF01 was deposited in GenBank under accession number OQ848592.

### Phylogenetic analyses

Phylogenetic analysis was performed using the major capsid protein the subfamily *Acinetobacter* and phage terminase large subunit genes. The multiple alignments were obtained with the Clustal W algorithm. Phylogenetic trees were constructed by MEGA X software using the neighbor-joining method with a bootstrap of 1000 (Kumar et al., 2018).

### *Galleria mellonella* larvae infection model

*Galleria mellonella* wax moth worms have been used as a good model to evaluate the activity and toxicity of novel antimicrobial agents against *A. baumannii* (Tao et al., 2021). Before the experiments, *Galleria mellonella* larvae were starved for 24 h at 37°C in a 90-mm Petri dish under darkness. Fifty larvae (200–250 mg) were randomly selected to be sterilized with 75% alcohol before injection. Larvae were infected with 10 μL of CRAB (1 × 10^8^ CFU/mL) on the right side of the last proleg using a 10-μL microliter syringe (Shanghai GaoGe Industry and Trade Co., Ltd), and 10 μL of PBS was injected as a control, with ten larvae in each group. To test the efficacy of phage treatment, after 30 min, larvae were injected with 10 μL of phage suspension in SM buffer at an MOI of 0.001 into the last right proleg, and 10 μL of SM buffer was injected as the control. The survival of *Galleria mellonella* larvae was monitored at 8-h intervals for a period of 48 h, and they were cultured in the dark at 37°C. Larvae were considered dead when they did not respond to touching a syringe needle. The above experiments were repeated three times.

### Phage efficacy in the mouse infection model *in vivo*

To evaluate the efficacy and safety of the phage *in vivo*, 60 mice were intraperitoneally injected with 100 μL of CRAB (1 × 10^8^ CFU/mL) and 100 μL of PBS was injected as a control, with ten mice per group. Two hours post-infection, 100 μL of a single phage Ab_WF01 (at an MOI of 0.001) or imipenem (IPM; 5 mg/mL; Shanghai Yuanye Bio-Technology Co., Ltd., China) was intraperitoneally injected into the experimental group, and 100 μL of SM buffer was injected as a control (Leshkasheli et al., 2019). As a control group, the mice were challenged with PBS and received only phage. All mice used in the experiments were immunized via the intraperitoneal (i.p.) route using cyclophosphamide (200 mg/kg; Beijing Huamaike Biotechnology Co., Ltd., China) at 48 h intervals. The mortality of the mice in each group was observed daily for 7 days. For cytokine analysis, blood was sampled from the hearts of the mice immediately after they were euthanized on day 1. The serum supernatants were stored at − 80°C for cytokine analysis. The serum concentrations of tumor necrosis factor-α (TNF-α) and interleukin 6 (IL-6) were detected using ELISA kits (Solarbio, Beijing, China) according to the manufacturer’s instructions.

### Bacteria clearance and phage counting

To determine bacterial counts in mice on day 3 following CRAB infection, organ samples from each group, including the lung, liver, and spleen, were collected immediately after euthanasia. Each organ was homogenized with 1 mL of PBS and serially diluted in PBS. The titers of bacteria and phages in organ homogenates were determined using plate counting and double-layer agar assays, respectively.

### Histology of mouse organ tissue

To evaluate histological features, mice were sacrificed on day 3, and various organs (lung, liver, and spleen) were collected immediately. Those specimens were fixed in 4% paraformaldehyde for 24 h, were dehydrated in graded alcohol, and embedded in paraffin. Tissue sections (3 μm thick) were stained with H&E. Pathological changes in the organs of mice were observed using an optical microscope.

### Statistical analysis

All data were analyzed using the GraphPad Prism 8.0 software (GraphPad, Inc., San Diego, CA, USA). The significance of differences between two groups was determined using unpaired *t*-tests, and the significance of differences between multiple groups was determined using ANOVA. Values were considered statistically significant at *p* < 0.05.

## Results

### Phage isolation, host range, and morphology

**Figure 1:**
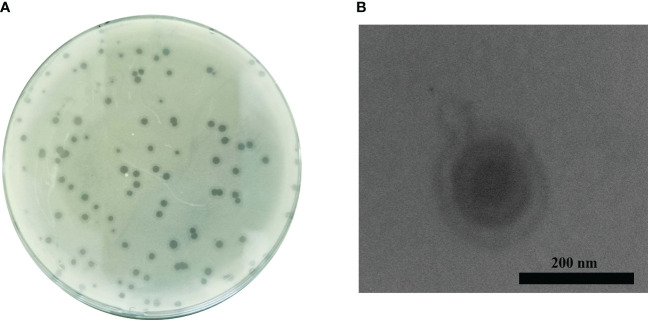
Isolation and morphology of phage Ab_WF01. **(A)** Plaque morphology of phage Ab_WF01 on the double-layer agar plate. **(B)** The morphology of phages Ab_WF01 was observed by TEM. Phages were negatively stained with 2% phosphotungstic acid. Scale bar represent 200 nm.

Phage Ab_WF01 was isolated from water samples in hospital wastewater using the CRAB clinical isolate as the host bacterial strain. The phage formed uniform round clear plaques 2-3 mm and plaque-surrounding halos were observed on double-layer plates (**Figure 1A**). The host range of phage Ab_WF01 was assessed using CRAB, CRPA, *K. pneumoniae*, MRSA, MDR *E. faecalis*, and CREC. Phage Ab_WF01 only lysed CRAB, and did not show any lytic activity against other five isolates (**Table 1**). EOP value is classified as high. Electron micrographs of phage Ab_WF01 were obtained by TEM, showing that the phage contained a hexagonal head with a diameter of approximately 85 nm and a short noncontractile tail with a length of approximately 40 (**Figure 1B**).

**Table 1:** Host range and EOP of phage Ab_WF01.

Strain	Phage Ab_WF01	
	Lytic Activity	EOP
CRAB	+	++
CRPA	–	–
*K. pneumoniae*	–	–
MRSA	–	–
MDR *E. faecalis*	–	–
CREC	–	–

+ was able to produce lytic zone, - was unable to produce lytic zone. EOP efficiency of plating, which was determined by calculating the ratio of plaque-forming units (PFUs) of each phage-susceptible strain to the PFUs of indicator strain. “++”: EOP > 1; “+”: EOP > 0.1; “-”: EOP =0.

### Analysis of the phage adsorption rate and one-step growth curve

**Figure 2:**
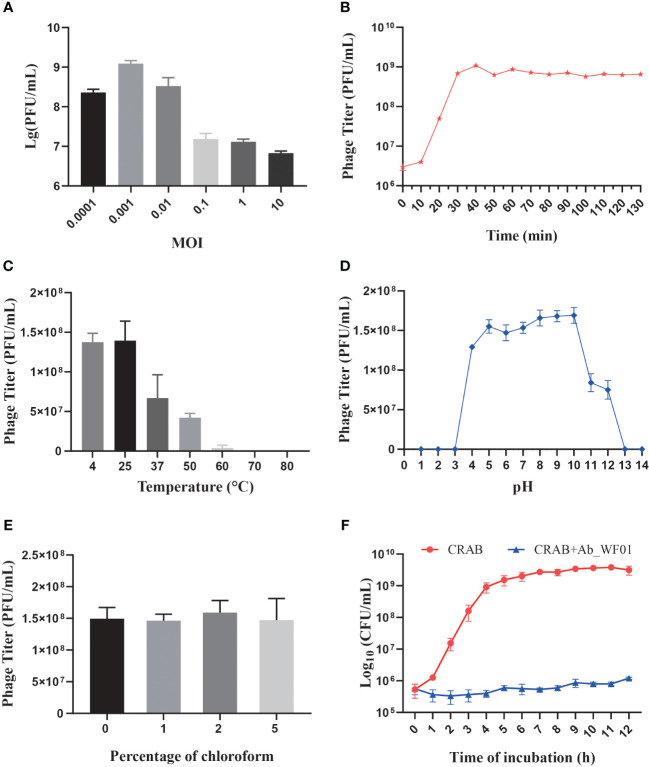
Biological characterization of Phage Ab_WF01. **(A)** The optimal MOI of phage Ab_WF01 is 0.001. **(B)** One-step growth curve of phage Ab_WF01 to host bacterial strain. **(C)** Thermal stability of phage Ab_WF01. The phages were incubated at different temperatures for 30 min. **(D)** Stability of phage Ab_WF01 at different pH values. **(E)** Sensitivity of phage Ab_WF01 to chloroform. The phage was treated with different volumes of chloroform for 30 min at 37°C. Phage titers were determined using the double-layer agar method. **(F)** Kinetics of lytic development of phage Ab_WF01 after infection of CRAB bacterial cells at a MOI of 0.001. The bacterial counts were measured every hour for 12 (h) The results are presented as the mean ± SD from triplicate biological replication.

To evaluate the lowest concentration at which phage Ab_WF01 could effectively inhibit host cell growth, CRAB was infected with Ab_WF01 at different MOIs, and the titer of the phage was determined using the double-layer agar assay. The results showed that when the MOI was 0.001, the phage titer reached its highest level (2 × 10^8^ PFU/mL) (**Figure 2A**). Therefore, an MOI of 0.001 was chosen as the standard dose for subsequent experiments.

The latent period and burst size of phage infection were evaluated through the one-step growth curve assay, and the results indicated a latent period of approximately 10 min. The ratio of liberated phage to originally infected bacteria during the latent period was approximately 151 PFU per infected cell, which was calculated as the burst size of the phage (**Figure 2B**).

### Thermal and pH stability

To evaluate the stability of phage Ab_WF01, the phage was treated at various temperatures and pH values to evaluate its stability. The phage showed the highest viability at 25°C, and its stability decreased as the temperature increased. When incubated at 37°C, the phage titer decreased to approximately 35% and completely no viability when the temperature reached 70°C (**Figure 2C**). For the pH stability test, the phage showed optimum activity in the pH range of 5–10. Further increases or decreases in pH resulted in reduced activity of phage Ab_WF01, sharply lost at pH = 11 and 12, and there was no activity at pH 1, 2, 3, 13, and 14 (**Figure 2D**).

### Chloroform sensitivity

The protein shell of phages contains lipids that play a crucial role in the delivery of the viral genome into the host cell (Peralta et al., 2013). To test whether phage Ab_WF01 virions contained lipids, the phage was treated with chloroform (0%, 1%, 2%, or 5%, *v/v*) at 37°C for 30 min. There were no significant changes in phage Ab_WF01 viability after exposure to different chloroform concentrations (**Figure 2E**). The results showed that lipid content was not present in the phage particles, and the phage was found to tolerate chloroform.

### Lytic activity

The lysis kinetics of phage Ab_WF01 against CRAB were measured every hour using a turbidity assay. In the control, the bacterial counts of the uninfected phage bacterial culture increased rapidly after 9 h of incubation, and then entered the stationary phase. However, bacterial growth was significantly inhibited when infected with phage Ab_WF01 compared to that in the control group (**Figure 2F**), suggesting a high bacteriostatic efficiency of phage Ab_WF01 against CRAB.

### Whole-genome analysis

**Figure 3:**
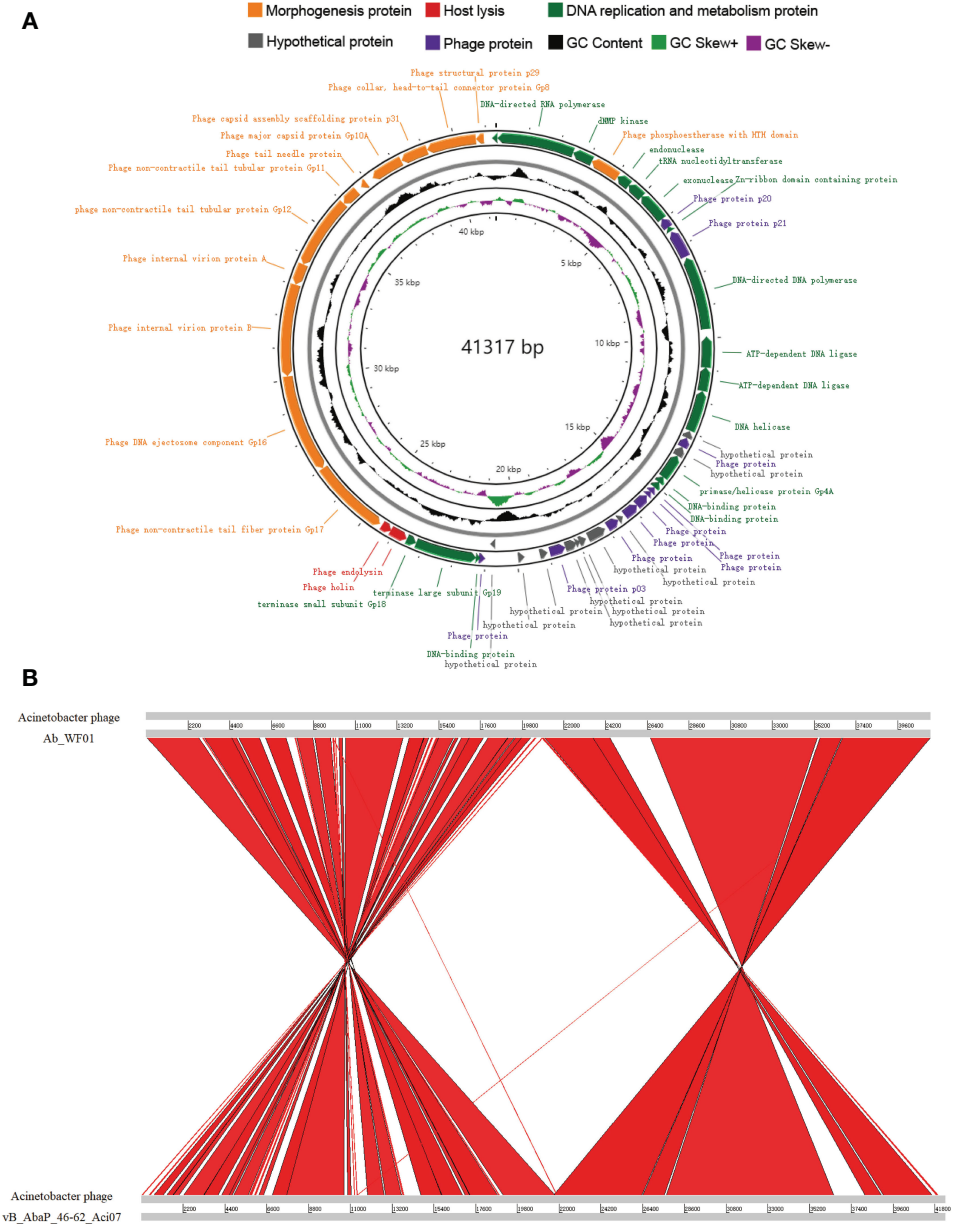
Genome characteristics and comparative analysis of phage Ab_WF01. **(A)** Genome map of phage Ab_WF01. Circular genome visualization of Ab_WF01 was constructed using the CGView server. The innermost ring presents GC skew (+, green; −, purple), and the black ring in the middle represents the GC content. The two outermost circle indicate the predicted ORFs of phage Ab_WF01 along with the direction of transcription indicated with arrows. Different color-coded CDSs represent different functions: morphogenesis protein (orange), Host lysis (red), DNA replication and metabolism protein (green), hypothetical protein (gray), and phage protein (purple). **(B)** Genome comparative analysis of *A baumannii* phage Ab_WF01 and phage vB_AbaP_46-62_Aci07. Comparison was carried out using the Artemis Comparison Tool (ACT).

The whole genome sequence of phage Ab_WF01 revealed a size of 41, 317 bp and GC content of 39.12% (**Figure 3A**). Fifty-one open reading frames (ORFs) were found in the phage Ab_WF01 genome. Coding density of ORFs was 93.93%, covering a total of 38, 808 bp. The maximum length ORF was 3099 bp and the minimum length was 114 bp, and these encoded phage DNA ejectosome components and hypothetical proteins, respectively. Nucleotide sequence of the genes encoding tRNA in the genome of phage Ab_WF01 have not been reported so far. Moreover, there were no antibiotic resistance or virulence genes in the phage Ab_WF01 genome, indicating that this phage has little ability to mediate the horizontal transfer of antibiotic resistance genes. Approximately 80.4% (41 out of 51) of the predicted ORFs were identified as known putative functional proteins by searching BLASTX against the NCBI database. Additionally, 10 other ORFs were not matched with the proteins in the databases and were classified as hypothetical proteins. The Ab_WF01 functional gene annotations were predicted to belong to five groups: proteins involved in DNA replication/modification, metabolism, lysis proteins, DNA packaging, and phage structural-related proteins.

The similarity of phage Ab_WF01 with other phages was comparatively analyzed using BLAST in the NCBI database, revealing that *Acinetobacter* phage vB_AbaP_46-62_Aci07 (GenBank number: NC_048076.1) shared the highest similarity with phage Ab_WF01, which showed 93.10% identity with 88% query coverage. To determine the homologous region in the genome of phage Ab_WF01 and the phage with the highest similarity, phage vB_AbaP_46-62_Aci07, we aligned the whole genome sequence of the two phages using the Artemis Comparison Tool (ACT). The average nucleotide identity (ANI) between the genomes of Ab_WF01 and vB_AbaP_46-62_Aci07 was 91.87% (calculated using ANI, http://enve-omics.ce.gatech.edu/ani/). Comparative genomic analysis showed that the genome sequence of Ab_WF01 was highly homologous to that of vB_AbaP_46-62_Aci07, and there were several large-scale inversions in the chromosomes associated with DNA replication and phage structure (**Figure 3B**).

### Phylogenetic analysis

**Figure 4:**
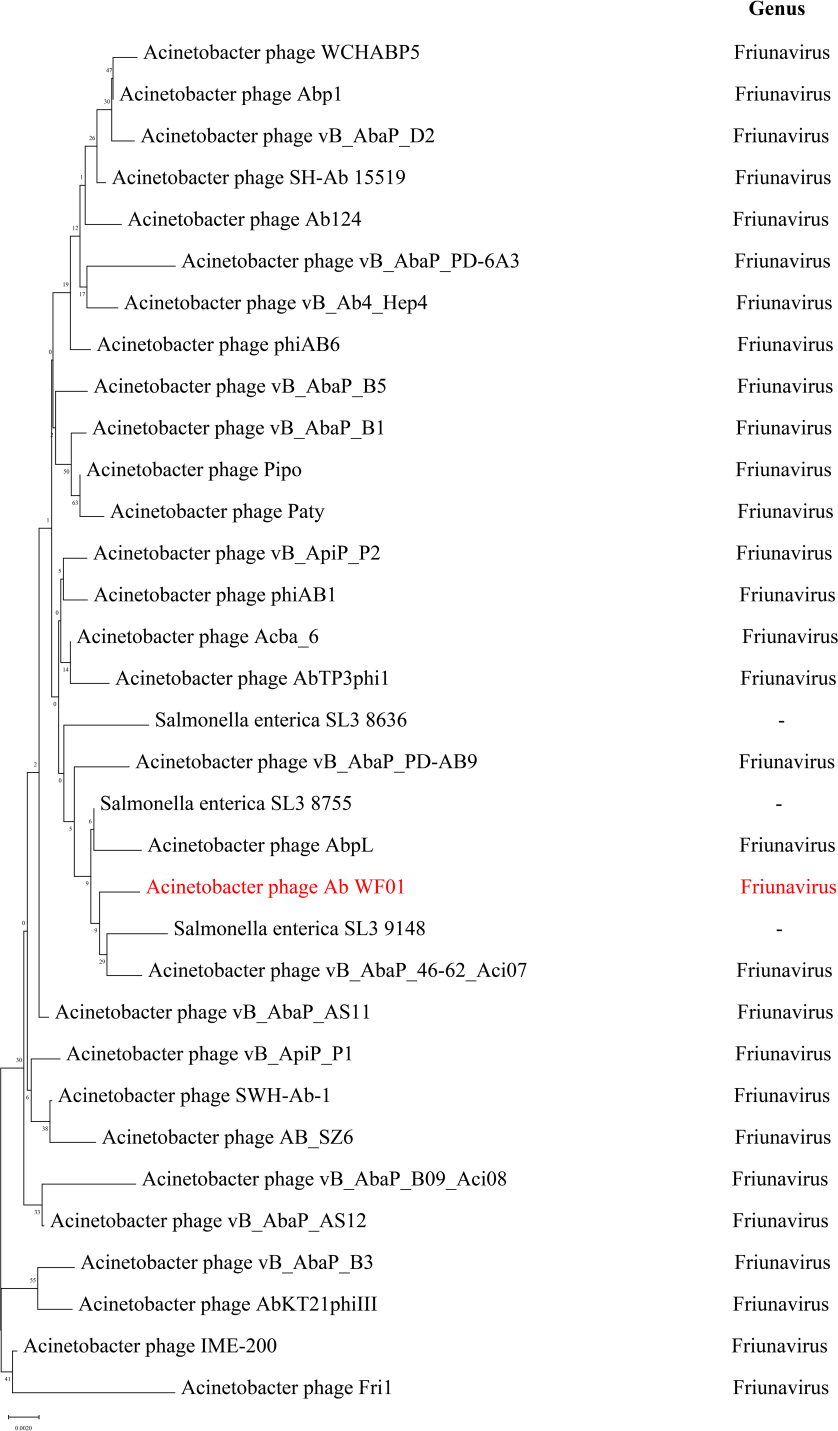
Phylogenetic trees of phage Ab_WF01 large subunit terminase. The trees were generated using MEGA X by Neighbor-joining method with 1000 bootstrap replicates. The phages of genus *Friunavirus* are shown in right. The phage Ab_WF01 is marked in red.

Phylogenetic trees were constructed using the terminase large subunit protein to infer the evolutionary relationships between Ab_WF01 and other phages (**Figure 4**). The terminal large subunit is generally considered the most conserved gene in phages and is commonly used for evolutionary analysis in phage classification. As shown in **Figure 4**, Ab_WF01 was clustered with *Acinetobacter* phage vB_AbaP_46-62_Aci07 and *Acinetobacter* phage AbpL, which belong to the genus *Friunavirus*, subfamily *Beijerinckvirinae*, family *Autographiviridae*, and order *Caudovirales*. According to the current guidelines of the International Committee on Taxonomy of Viruses (ICTV), phage Ab_WF01 is a new species of *Friunavirus* genus phage.

### *In vivo* efficacy of phage Ab_WF01 in *Galleria mellonella* model

**Figure 5:**
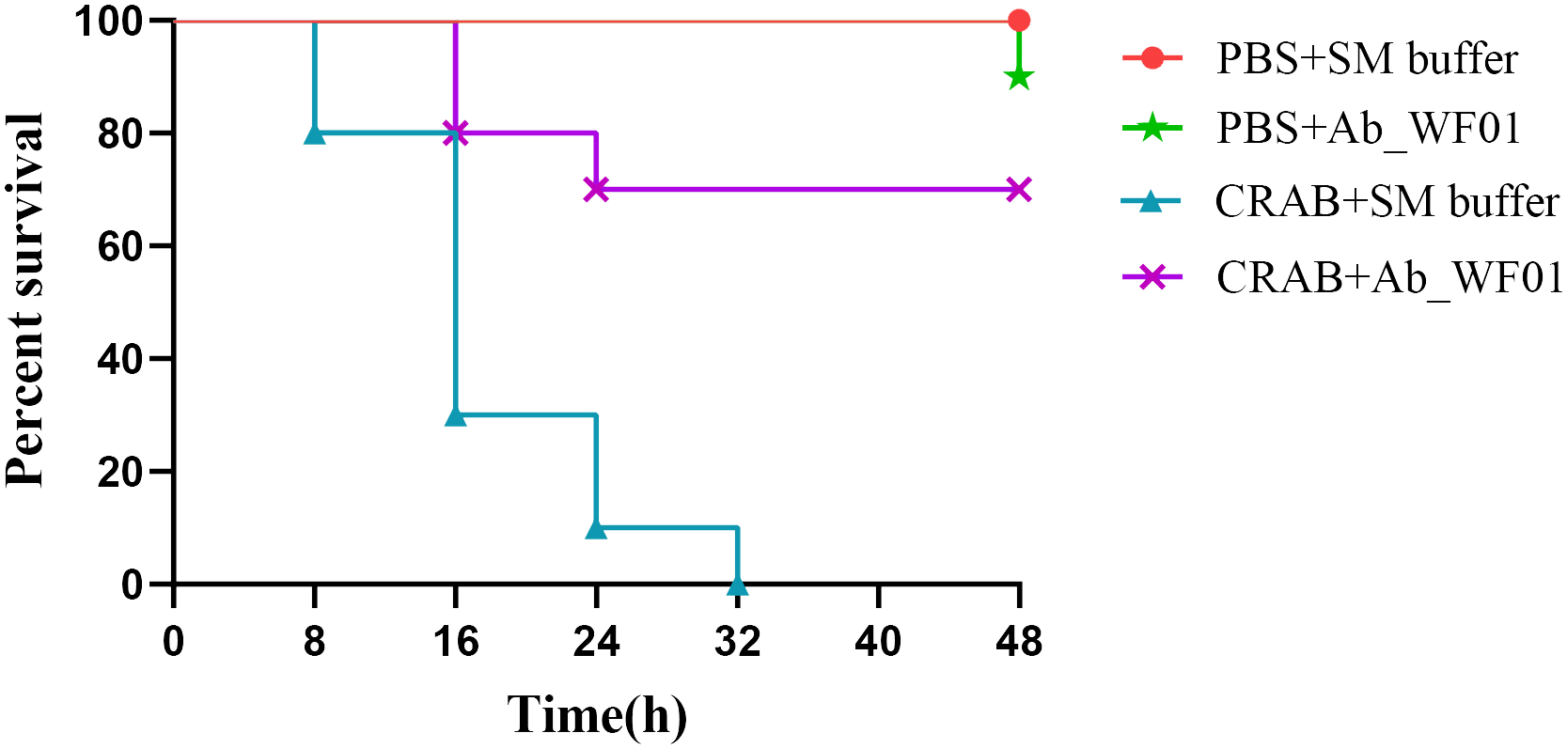
Survival curves of *G. mellonella* larvae treated with phage Ab_WF01. *G. mellonella* larvae were injected with 10 μL CRAB or PBS (negative control) in the second to last right proleg. After 30 min, each larva was treated with 10 μL phage Ab_WF01 with a MOI of 0.001 or SM buffer (phage control). The survival of larvae was scored every 8 h for 48 h. Ten larvae were examined in each group and experiment was repeated three times. The survival rates were plotted using the log-rank Mantel-Cox test. Data were analyzed using GraphPad Prism 8.

To preliminary evaluate the efficacy of phage Ab_WF01 against CRAB infection *in vivo*, we used *Galleria mellonella* larvae as infection model. As shown in **Figure 5**, all larvae in the CRAB infection group died at 32 h, whereas the mortality of the larvae was reduced by phage treatment. Compared to the untreated control group, larvae treated with phage showed a statistically significant improvement in survival rates at 48 h (p < 0.01). There was no significant difference in survival between the phage control group and the buffer (PBS + SM) control group, suggesting the safety of phage Ab_WF01. The survival rate of the phage control group was the same as that of the buffer (PBS+SM) control group, indicating the safety and feasibility of phage Ab_WF01.

### *In vivo* efficacy of phage Ab_WF01 in a mice CRAB infection model

**Figure 6:**
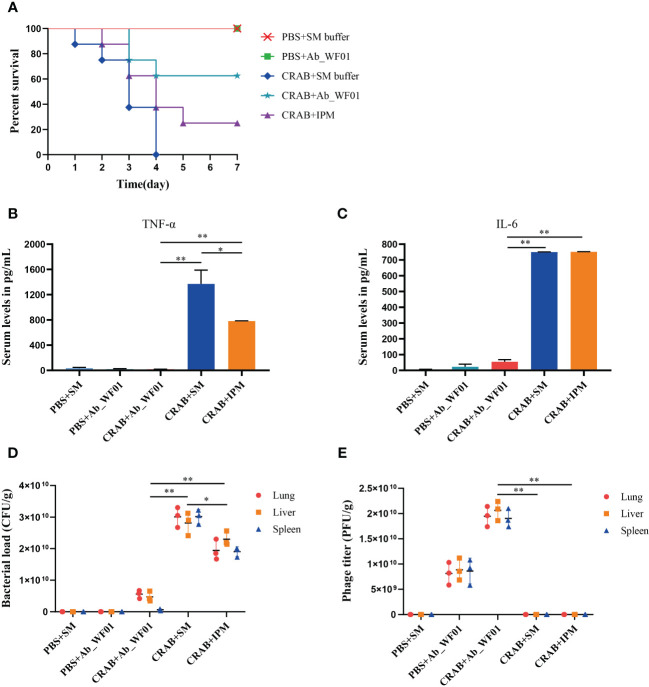
Evaluation of efficiency of phage Ab_WF01 using a mice model *in vivo*. Mice were intraperitoneally injected with 1 × 10^7^ CFU/mouse CRAB, and PBS was injected as uninfected control. After 1 h post-infection, they were treated with phage Ab_WF01 (MOI = 0.001 and phage control), SM buffer (vehicle control) or IPM (5 mg/mL) by intraperitoneal administration. The control group was administered with SM buffer under the same conditions. Mice were euthanized 3 days after i.p. and lung, liver, and spleen tissue were harvested. **(A)** The survival rates of mice were monitored each day for 7 days. The **(B)** TNF-α and **(C)** IL-6 cytokine levels in serum of mice were measured at 3 days after administration using cytokine ELISA (n=3). **(D)** Bacterial load and **(E)** phage titer in mouse organs. The lung, liver, and spleen of mice from each group were harvested and homogenized 3 days after treatment. The bacterial CFU in the lung, liver, and spleen was quantified by ten-fold serially diluted tissue homogenates. The titer of phage Ab_WF01 were measured using the double-layer agar method. Asterisks are defined as: * *p* < 0.05, ** *p* < 0.01.

To further assess the efficacy and safety of phage Ab_WF01 *in vivo*, we evaluated the survival rate, histological features, number of bacteria and phages in the lung, liver, or spleen, and immunogenicity through intraperitoneal injection of phage against CRAB. As shown in **Figure 6A**, all mice infected with CRAB died within four days post-infection. In contrast, 60% of mice infected with the bacteria, followed by treatment with phage, survived. After five days of treatment, the survival rate of the phage treated group was 60%, which was higher than the antibiotic treated group (30%). Furthermore, all mice in the negative control group (injected with PBS or Ab_WF01) remained alive after seven days. Mice in the phage treated group had better efficacy than those in the IPM treated groups, suggesting that phage treatment could rescue CRAB-infected mice.

To determine if serum inflammatory cytokines might be important in phage treatment, we next checked the levels of TNF-α and IL-6 in mouse serum after phage treatment (**Figures 6B, C**). We observed that serum TNF-α and IL-6 levels were significantly higher in the bacteria-only treated group than in the phage treated group, indicating that phage treatment may reduce the pro-inflammatory response caused by CRAB infection alone. In contrast to the IPM group, the serum TNF-α levels was significantly reduced after phage treatment (*p* < 0.01). In addition, the levels of TNF-α and IL-6 in the phage-only treated group were compared to those in mice treated with SM buffer or phage alone, but IPM-treated mice were higher than those in the phage-treated group. The results showed that phage Ab_WF01 had a good substitution effect on the IPM.

The death of mice with CRAB infection could be rescued by phage Ab_WF01 treatment, and we reasoned that phage could reduce bacterial load. To confirm this, the concentrations of bacteria and phages in the lungs, liver, and spleen of mice were tested for 3 days after treatment. In the bacteria-only treated group, the bacterial loads of the mouse lung, liver, and spleen were significantly higher than those in the other treated groups (**Figure 6D**). Compared to the untreated group, the bacterial load was lower in mice in the IPM-treated group and much lower in mice in the phage-treated group. Additionally, the load in the phage therapy group was not significantly different from that in the control group. These results suggest that phage Ab_WF01 has a better bacterial clearance effect than that of IPM. Next, we measured the phage titer in the lungs, liver, and spleen of mice, as shown in **Figure 6E**. The phage titers in the lung, liver, and spleen of mice in the phage treated group were significantly higher than those in the bacteria alone group and IPM treated group (*p* < 0.01), and no phages were detected in the IPM group and bacteria-only treated group and the buffer only group. These results are consistent with the fact that the bacterial load and use of phage Ab_WF01 therapy resulted in a decrease in the number of bacteria.

**Figure 7:**
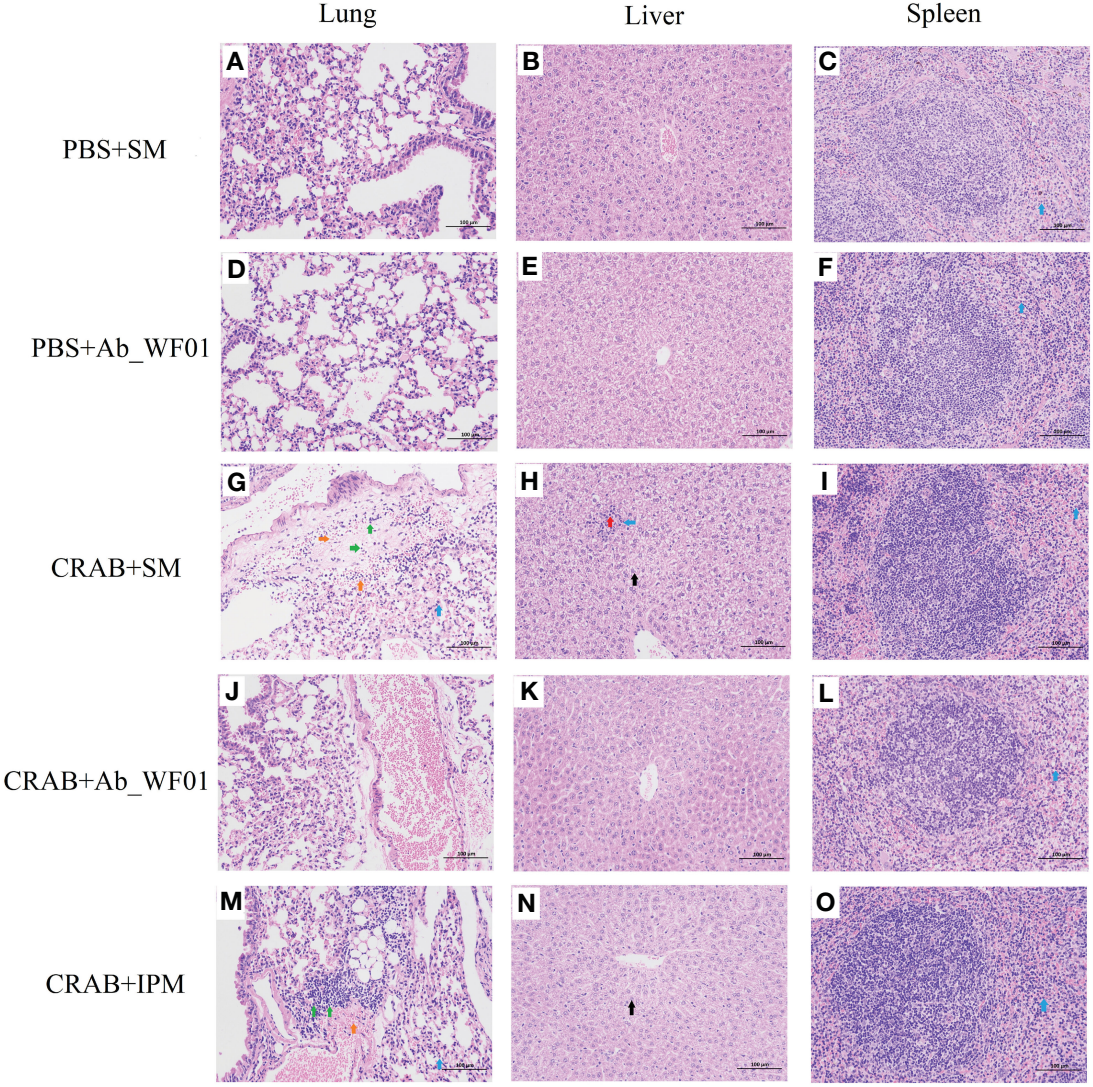
Histopathology features of the main organs from different groups of mice. The lung **(A, D, G, J, M)**, liver **(B, E, H, K, N)**, and spleen **(C, F, I, L, O)** from the mice challenged with phage were removed and immediately placed in 4% paraformaldehyde. Tissue sections were stained with H&E and then observed in a microscope at a magnification 200х. Blue arrow: with a small amount of granulocyte infiltration; Green arrow: multiple perivascular edema with a small amount of lymphocytic infiltration; Orange arrow: multifocal hemorrhage is visible in the tissue; Red arrow: hepatocyte necrosis in the hepatic parenchyma; Black arrow: extensive hepatocyte degeneration in the central vein, around the portal area and within the hepatic parenchyma.

To assess the safety of phage Ab_WF01 on CRAB-infected mice *in vivo*, histological changes in lung, liver, and spleen samples were analyzed using H&E staining (**Figure 7**). The histopathological analyses of the organs from the phage control and buffer control groups did not reveal any significant pathological findings, which indicates that tissue cells are of normal structure. Local perivascular edema with bleeding and lymphocyte distribution was seen around the bronchioles of individuals treated with bacteria alone and IPM alone (**Figures 7G, M**). A small amount of granulocyte infiltration was observed in the spleen of all groups. In addition, there was marked hepatocyte necrosis, condensed nuclear debris, and partial granulocyte infiltration in the liver (**Figure 7H**). However, most of these pathological findings were markedly improved in the organs of the phage-treated group (**Figures 7J–L**), indicating that phage Ab_WF01 has efficacy potential against CRAB infection.

## Discussion

Antibiotic resistance is considered to be one of the major challenges to public health worldwide, especially with the emergence of multi-drug resistant (MDR) bacteria, leading to a lack of effective antibiotic therapy in clinical practice. For example, an increase in carbapenem resistance is a major problem, which is considered a last-resort antibiotic treatment against infections with multidrug-resistant gram-negative bacteria (Papp-Wallace et al., 2011). Carbapenem-resistant *A. baumannii* (CRAB) infections and drug resistance rates are increasing because these strains have developed resistance to most clinically general antibiotics (Ibrahim et al., 2021). In recent years, the discovery and development of new antibiotics have slowed as bacteria acquire resistance to antibiotics at an unforeseen rate. A novel antimicrobial strategy is required to prevent this threat. The use of phages in the treatment of CRAB infections is particularly important.

Although phage therapy is considered a potential alternative treatment for CRAB, further research is needed to unravel some unanswered questions, especially regarding its *in vivo* applications before possible routine clinical use. In this study, we successfully isolated phage Ab_WF01, which belongs to *Autographiviridae* family and has a short noncontractile tail based on its morphological features. Moreover, Ab_WF01 is similar to other *Autographiviridae Acinetobacter* phages (Li et al., 2023), as verified by electron microscopy and bioinformatics analysis. Interestingly, the tail terminal of Ab_WF01 was longer than that of other *Autographiviridae* family phages. We speculated that the structure might be associated with phage depolymerase because the phage tail structure is associated with phage adsorption to hosts (Ackermann, 2009). Furthermore, phage Ab_WF01 forms clear round plaques with a halo, which are associated with phage-derived depolymerases on agar plates (Vukotic et al., 2020). This phenomenon confirmed that depolymerase has antibiofilm activity, which can degrade the capsular exopolysaccharides of bacteria.

We next investigated the physiological characteristics of phage Ab_WF01 (**Figure 2**). The burst size and latent period are key factors to prioritize in phage therapy (Gao et al., 2022). In general, phages with high burst sizes and short latent periods are more effective at lysing bacteria (Abedon and Culler, 2007). Phage Ab_WF01 has a high adsorption rate and large burst size. The pH and temperature of phage storage are important factors that affect phage activity and stability (Wintachai et al., 2020). Phage Ab_WF01 showed activity over a wide temperature range allows it to withstand temperatures as high as 60°C. Similar to *Acinetobacter* phage TCUP2199 (Mardiana et al., 2022), Ab_WF01 lost its lytic activity at 70°C. The pH stability showed that phage Ab_WF01 is more tolerant to a strong basicity in the pH activity range of 4-12 compared to *Acinetobacter* phage vB_AbaP_PMK34 (Abdelkader et al., 2022). Furthermore, phage Ab_WF01 was very stable after treatment with different concentrations of chloroform. Phage activity and stability are essential conditions for the preparation of phage agents (Pires et al., 2020), which makes it a potential biocontrol agent in the future. Moreover, the lytic activity of phage testing showed that Ab_WF01 strongly inhibited bacterial growth in a dose-dependent manner. It is evident from these results that the phage Ab_WF01 showed a high level of lytic activity against CRAB *in vitro*, indicating its potential for use of this phage as an antibacterial agent.

The safety and novelty of phages can be predicted by bioinformatic analysis and used for phage therapy (Pu et al., 2022). We found no antibiotic resistance or virulence genes in the genome of phage Ab_WF01, indicating that those genes of phage Ab_WF01 have not been reported so far. Following the evolutionary relationship of phage Ab_WF01 with other related phages, we constructed a phylogenetic tree based on the terminase large subunit. Phylogenetic analysis showed that phage Ab_WF01 shared the closest evolutionary relationship with *Acinetobacter* phage vB_AbaP_46-62_Aci07, a member *of the Podoviridae* family. A comparative analysis with vB_AbaP_46-62_Aci07 revealed that Ab_WF01 is a novel lytic phage belonging to the *Podoviridae* family, which is consistent with previous phylogenetic analysis results. These features suggest that it can be used as a candidate biocontrol agent (Hyman, 2019).

*Galleria mellonella* has been widely used as a good model to study host-pathogen interactions because it is cheap and poses few ethical concerns compared to other animal models (Tao et al., 2021). Thus, we first applied the *G. mellonella* larval infection model to assess the efficacy of phage Ab_WF01 against CRAB. We found that phage Ab_WF01 had better efficacy in CRAB-infected *G. mellonella* larvae *in vivo*. This finding is consistent with previous reports that a single phage enhances the survival rate of *G. mellonella* against *A. baumannii* (Jeon et al., 2019). To further explore the potential of phage Ab_WF01 for clinical application, we evaluated *in vivo* the efficacy of phage Ab_WF01 in a C57BL/6j mouse model. The results revealed that the CRAB-infected mice treated with phage Ab_WF01 exhibited an approximately 2-to 3-fold higher survival rate than that of IPM treatment, indicating that phage Ab_WF01 has a certain the efficacy in CRAB-treated mice. This is consistent with our *in vivo* studies on the efficacy of phages on the *G. mellonella* larvae infection model. Because mice and *G. mellonella* larvae have different immune systems (Tao et al., 2021), we further assessed the safety of phage Ab_WF01 against CRAB. The cytokine results suggest that CRAB caused an inflammatory response in BALB/c mice, which could be rescued by phage Ab_WF01 treatment. In addition, histopathological examination of various mouse organs (lung, liver, and spleen) showed that phage Ab_WF01 effectively alleviated lesions caused by CRAB, supporting the serum cytokine results. We used lower doses of Ab_WF01 phages (MOI = 0.001) to treat CRAB infection in an *in vitro* model, which can achieve effects similar to those previously reported for high dosages of Bϕ-R2096 phage (MOI = 10) (Jeon et al., 2019). This means that phage therapy titers that are more flexible than higher phage titers may be more effective. To the best of our knowledge, successful phage treatment of CRAB infection in humans has been reported less frequently (Tan et al., 2021; Wu et al., 2021). These results indicate that phage Ab_WF01 can be used as an alternative drug to replace antibiotic clearance for CRAB infection and has potential clinical applications.

In conclusion, we describe phage Ab_WF01 as a new lytic phage that is effective against CRAB infection. According to the physiological characteristics, comprehensive genomic analysis, and evaluation of the efficacy in *G. mellonella* larvae and C57BL/6j mice, the phage Ab_WF01 may have potential clinical applications in the treatment of CRAB infections.

## Data availability statement

The datasets presented in this study can be found in online repositories. The names of the repository/repositories and accession number(s) can be found in the article/supplementary material.

## Ethics statement

The animal study was approved by Ethical Committee of Animal Experiments of Weifang Second Medical University. The study was conducted in accordance with the local legislation and institutional requirements.

## Author contributions

ZW: Writing – original draft, Writing – review & editing. XY: Conceptualization, Writing – review & editing. HW: Data curation, Writing – review & editing. SW: Formal analysis, Writing – review & editing. RF: Validation, Writing – review & editing. XL: Methodology, Writing – review & editing. JX: Software, Writing – review & editing. QW: Investigation, Writing – review & editing. ZL: Formal analysis, Writing – review & editing. NS: Writing – review & editing.
